# Quantitative Functional Arterial Spin Labeling (fASL) MRI – Sensitivity and Reproducibility of Regional CBF Changes Using Pseudo-Continuous ASL Product Sequences

**DOI:** 10.1371/journal.pone.0132929

**Published:** 2015-07-14

**Authors:** Rebecca M. E. Steketee, Henri J. M. M. Mutsaerts, Esther E. Bron, Matthias J. P. van Osch, Charles B. L. M. Majoie, Aad van der Lugt, Aart J. Nederveen, Marion Smits

**Affiliations:** 1 Department of Radiology, Erasmus MC–University Medical Center Rotterdam, Rotterdam, the Netherlands; 2 Department of Radiology, Academic Medical Center Amsterdam, Amsterdam, the Netherlands; 3 Biomedical Imaging Group Rotterdam, Departments of Medical Informatics and Radiology, Erasmus MC–University Medical Center Rotterdam, Rotterdam, the Netherlands; 4 C.J. Gorter Center for High Field MRI, Department of Radiology, Leiden University Medical Center, Leiden, the Netherlands; Leibniz Institute for Neurobiology, GERMANY

## Abstract

Arterial spin labeling (ASL) magnetic resonance imaging is increasingly used to quantify task-related brain activation. This study assessed functional ASL (fASL) using pseudo-continuous ASL (pCASL) product sequences from two vendors. By scanning healthy participants twice with each sequence while they performed a motor task, this study assessed functional ASL for 1) its sensitivity to detect task-related cerebral blood flow (CBF) changes, and 2) its reproducibility of resting CBF and absolute CBF changes (delta CBF) in the motor cortex. Whole-brain voxel-wise analyses showed that sensitivity for motor activation was sufficient with each sequence, and comparable between sequences. Reproducibility was assessed with within-subject coefficients of variation (wsCV) and intraclass correlation coefficients (ICC). Reproducibility of resting CBF was reasonably good within (wsCV: 14.1–15.7%; ICC: 0.69–0.77) and between sequences (wsCV: 15.1%; ICC: 0.69). Reproducibility of delta CBF was relatively low, both within (wsCV: 182–297%; ICC: 0.04–0.32) and between sequences (wsCV: 185%; ICC: 0.45), while inter-session variation was low. This may be due to delta CBF’s small mean effect (0.77–1.32 mL/100g gray matter/min). In conclusion, fASL seems sufficiently sensitive to detect task-related changes on a group level, with acceptable inter-sequence differences. Resting CBF may provide a consistent baseline to compare task-related activation to, but absolute regional CBF changes are more variable, and should be interpreted cautiously when acquired with two pCASL product sequences.

## Introduction

Arterial spin labeling (ASL) perfusion magnetic resonance imaging (MRI) is being increasingly used for imaging of task-related brain activation. Such functional ASL (fASL) has been used to study the neural correlates of a multitude of cognitive domains, including attention [1], memory [2], language [3], visual [4] and sensorimotor processing [5], and is increasingly considered as an alternative to blood oxygen level-dependent (BOLD) functional MRI (fMRI), which has been predominantly used as a marker for neural activation during the last two decades.

ASL has several advantages over BOLD imaging with respect to acquisition and interpretation. First, ASL has better sensitivity in low frequency paradigms. The BOLD signal has been shown to be confounded by slow ‘drift’ effects in baseline signal, which are reduced in ASL imaging as a result of the pairwise subtraction of labeled and unlabeled images [5]. Second, despite the intrinsically low signal-to-noise ratio (SNR) of ASL, spatial localization of neuronal activity seems more accurate when measured with ASL than with BOLD. The BOLD signal is affected by macrovascular venous effects [6] whereas ASL is more sensitive to the microvasculature [7]. The interpretation of the BOLD signal is more complex as it reflects a combination of cerebral blood flow (CBF), cerebral blood volume (CBV) and cerebral metabolic rate of oxygen consumption (CMRO2) [8,9], whereas ASL provides a measure of CBF that is relatively less sensitive to other hemodynamic parameters. Furthermore, ASL provides an in principle quantitative measure of CBF, whereas the BOLD signal is relative. These advantages favor the application of fASL over fMRI BOLD for task-related brain imaging.

The quantitative aspect of ASL in particular could facilitate the comparison and exchange of CBF values across multiple sites and enable multicenter studies, for instance, to pool data. However, before fASL can be used as such, its variability needs to determined, not only within sessions and scanners, but also between product sequences of different vendors, as each vendor provides its own particular ASL implementation. Reproducibility of ASL in general is affected by intrinsic properties, such as low SNR and relative sensitivity to hemodynamics such as arterial transit time (ATT) [10]. In addition, although within-sequence reproducibility is sufficient for the commonly available labeling schemes [11–22], pseudo-continuous arterial spin labeling (pCASL) has been shown to be best reproducible within session, scanner, and vendor, being more stable and less variable than continuous ASL (CASL) and pulsed ASL (PASL) [23,24].

Nevertheless, not every user is aware of the potential impact of these factors and may assume that any ASL implementation will provide the same information, as can be expected from a quantitative technique. This may seem particularly appealing for the quantification of brain activation in functional imaging studies. The extent to which different vendor implementations affect these data is not known. We will therefore compare two pCASL product sequences as implemented by two different vendors, while limiting adjustment of sequence parameters to within the constraints imposed by the vendor-specific implementation.

Baseline or resting CBF values have been found to be well reproducible within sessions, within scanners, and between scanners of the same vendor on a whole-brain level, whereas on a regional level reproducibility was lower [23,24]. We previously assessed the reproducibility of whole-brain resting CBF within and between pCASL product sequences at 3T scanners of two different vendors [25]. Mean global CBF did not differ between product sequences, but voxel-by-voxel assessment revealed regional differences. Regional variability presents a challenge for fASL, where local effects are of particular interest. In addition to the variability in regional CBF changes, the variability in the detection of such CBF changes needs to be assessed. Sufficient and similar sensitivity to detect local task-induced CBF changes is a prerequisite for multicenter fASL implementations, and essential to good reproducibility.

As of yet, variability of quantitative fASL and variation of sensitivity for task-induced CBF changes between product sequences of different vendors have not been studied. Not only is this information essential for exchanging and comparing fASL data, but results generated by one product sequence can only be generalized to another if variability between them is known. The aim of the present study was to assess quantitative fASL by 1) assessing sensitivity to detect regional CBF changes in a voxel-wise whole-brain analysis, and 2) by investigating regional reproducibility of both resting CBF and task-induced CBF changes in the primary motor cortex, within and between pCASL product sequences from two major vendors. We investigated this by means of paced finger tapping, a simple behavioral paradigm that is known to elicit robust and consistent regional activation in the primary motor cortex in a multitude of activation studies using BOLD as well as fASL (e.g [5–7]). We employed this paradigm in healthy volunteers using product sequences from two different vendors.

## Methods

### Participants

Twenty-two healthy volunteers, aged 18–40 years, were recruited as part of a larger study on ASL reproducibility [25]. Participants were recruited through advertisement at the University of Amsterdam. Only participants with no history of neurological or psychiatric disease were included. Participants that used medication other than contraceptives, or had contraindications for MRI were excluded.

Participants were asked to limit their consumption of alcohol, nicotine and caffeine to a maximum of three units 12–24 hours prior to scanning, and to refrain from consuming alcohol, nicotine and caffeine 12 hours prior to scanning. The study was approved by the local medical research ethics committees of both sites: the Erasmus MC–University Medical Center Rotterdam and the Academic Medical Center, Amsterdam and was conducted according to the Declaration of Helsinki. All participants gave written informed consent and received financial compensation for participation.

### Image acquisition

**Fig 1:**
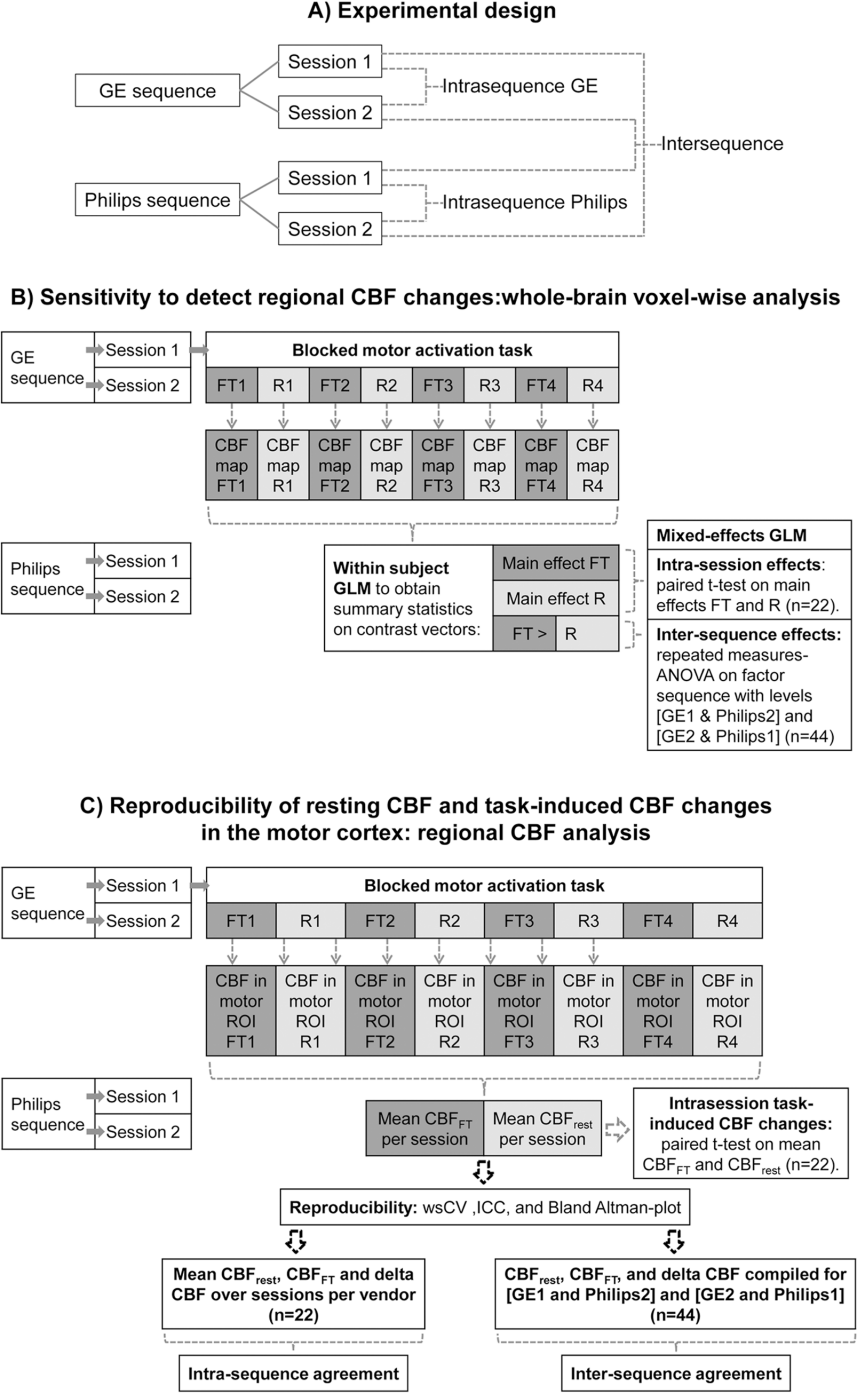
design and analyses. Schematic overview of A) experimental design and analyses of B) whole-brain voxel-wise activation sensitivity and of C) reproducibility of regional resting CBF and regional task-induced CBF changes.

Imaging was performed on a 3T Intera (Philips Healthcare, Best, the Netherlands) and a 3T Discovery MR750 (GE Healthcare, WI, USA) scanner, using an 8 channel receive head coil. Participants were scanned twice on both scanners, i.e. four sessions in total, in no specific order (Fig 1A). Scanning sessions were separated by at least one week, but no more than four weeks.

A high resolution 3D T1-weighted (T1w) scan for anatomical reference was acquired during one of the two sessions on each scanner. Perfusion data were acquired using pCASL product sequences that were provided by the vendor of each scanner. As we aimed to assess the reproducibility of the currently implemented (i.e. product) sequences, we chose to employ vendor-supplied sequences rather than to reprogram the sequences to make them match completely. Hence, parameters were only adjusted within the given limits of the clinical scanning sequences. Imaging data of the two sites were acquired by two different researchers. Strict agreements were made about the complete process of instructing and positioning participants in order to minimize differences between researchers and subsequently between sequences. Details of both pCASL sequences are listed in Table 1. Note that on the GE scanner a segmented 3D readout was employed, whereas on the Philips scanner single-shot multi-slice 2D imaging was combined with averaging to obtain a temporal resolution similar to the GE-sequence. Another difference between sequences was that on the GE scanner perfusion data are averaged during acquisition, whereas for Philips data is averaged after acquisition, after pair-wise subtraction of label and control images during post-processing. As the GE sequence only provides three fixed post-labeling delays (1 025, 1 525 and 2 025 ms for respectively children, adults, and older adults or adults with cerebrovascular disease), a delay of 1 525 ms, considered most suitable for the current population, was selected for all ASL imaging.

**Table 1:** Vendor-specific parameters of the pCASL product sequences.

	GE	Philips
Readout sequence	3D FSE stack-of-spirals	2D gradient-echo single-shot EPI
Acquisition matrix	8 arms with 512 sampling points	80 * 80
Parallel imaging	No	SENSE factor = 2.5
Voxel size	3.75 * 3.75 * 4 mm^3^	3 * 3 * 7 mm^3^
Field of view (FOV)	24 * 24 cm^2^	24 * 24 cm^2^
Number of slices	36	17
Echo time	10.5 ms	17 ms
Repetition time	4 600 ms	4 000 ms
Flip angle	111°	90°
Labeling duration	1 450 ms	1 650 ms
Post-labeling delay	1 525 ms	1 525 ms
Labeling plane (distance from AC-PC line^a^ in head-feet direction)	89 mm	72 mm
Background suppression	Yes	Yes
Vascular crushers	No	No
Total scanning time	2:01 min	2:08 min
NEX (no. of excitations) GE; NSA (no. of repetitions) Philips	1	16

^a^ anterior commissure–posterior commissure line

### Motor activation paradigm

Eight ASL scans were acquired, during which participants performed a blocked motor activation task (Fig 1). Block length was equal to acquisition time of one scan, i.e. 2 minutes. Participants were instructed to tap the fingers of both hands to the thumbs in random order (finger tapping, FT) during the odd scans and to keep their hands still (rest) during even scans. FT was auditorily paced at a frequency of 1 Hz.

### Data processing

The imaging data were processed according to the methods described in full in Bron *et al*., 2014 [26].

#### Tissue segmentation

The unified tissue segmentation method [27] of SPM8 (Statistical Parametric Mapping 8, Wellcome Trust Centre for Neuroimaging, University College London, UK) was used to obtain gray matter (GM), white matter (WM) and cerebrospinal fluid (CSF) probability maps from the T1w image.

#### ASL post-processing

For Philips data, label and control pCASL images were pair-wise subtracted (without motion correction) and averaged to obtain perfusion weighted images. For GE data, the perfusion-weighted images, as provided by the scanner, were used. For each participant, the perfusion-weighted image and the GM probability map were rigidly registered (Elastix registration software [28]). The results of the registration were visually inspected and showed good correspondence.

#### Quantification

To quantify the perfusion-weighted maps of both pCASL sequences as cerebral blood flow (CBF) maps, a single-compartment model was used [10]:

$$CBF\;\left(ml/100g/min\right)=\frac{6\;000\;\lambda \;\Delta M\;e^{PLD/T_{1a}}}{2\;\alpha \;T_{1a}\;M_{0a}\;\left(1-e^{-\tau /T_{1a}}\right)}$$

Parameters used in this model and their values are summarized in Table 2. Differences in effective post-labeling delay for different slices resulting from the 2D multi-slice readout were accounted for in the Philips data (Table 2).

**Table 2:** variables of the single-compartment model used for quantification (based on Alsop *et al*., 2015 [10]).

Symbol	Variable	Value
*λ*	blood-brain partition coefficient for gray matter	0.9 mL/g
Δ*M*	perfusion-weighted image	Philips: corrected for transversal magnetization decay time (T_2_*) of arterial blood (50 ms) during the 17 ms echo time (TE) by e^TE/T2*^ [45]
*M*_0*a*_	equilibrium magnetization of arterial blood	GE: obtained by individual proton density maps, adjusted for T1 decay time of gray matter tissue (*T1*_*GM*_, 1.2 s) during saturation recovery time (*t*_*sat*_, 2 s) by 1 –*e* ^*-tsat/T1GM*^; Philips: scanner average (3.7*10^6^ a.u.) from previous study [46]
*PLD*	post-labeling delay	1 525 ms
*T*_1*a*_	longitudinal relaxation time of arterial blood	1 650 ms [47]
*α*	labeling efficiency	0.8 [48]. In order to correct for background suppression pulses [49]: for GEα* 0.75; for Philips: α* 0.83
*τ*	labeling duration	GE: 1 450 ms; Philips: 1 650 ms

### Whole-brain voxel-wise preprocessing and activation sensitivity analysis

Registered T1w images and CBF maps were transformed to a common template space based on the T1w images of all participants [26]; CBF maps were smoothed using an isotropic 8 mm full width at half maximum (FWHM) kernel.

Voxel-wise differences within and between sequences in relation to finger tapping (Fig 1B) were assessed using SPM8. Averaged CBF maps per block of finger tapping (FT) and rest were convolved with the hemodynamic response function and modeled on an individual level using a General Linear Model (GLM), yielding parameter estimates for the main effects of FT and rest and the contrast [FT > rest] which were subsequently used in group analyses (Fig 1B). Task-induced changes were assessed per session per sequence by pair-wise comparison of main effects of FT and rest for each participant during each session. As participants were scanned in a random order, we chose to assess inter-sequence differences by means of a repeated measures ANOVA on the contrast [FT > rest] for GE (session) 1 compared to Philips 2 and GE 2 to Philips 1 (n = 44). By comparing sessions this way, we can assume that temporal physiological variation affected intra-sequence and inter-sequence reproducibility to a similar extent. All voxel-wise results were thresholded at p<0.001 without correction for multiple comparisons, to be maximally sensitive to intra- and inter-sequence differences in the detection of CBF changes.

### ROI preprocessing and regional reproducibility analysis

#### ROI labeling and selection

Individual CBF maps were transformed to individual T1w image space for region of interest (ROI) analysis. ROIs for each participant were defined using a multi-atlas approach by registering thirty labeled T1w images, each containing 83 ROIs [29,30], to the participants’ T1w images, using a rigid, affine, and non-rigid model consecutively. For the current study, we focused on the bilateral primary motor cortex, i.e. the superior aspect of the precentral gyri, containing the hand-motor area [31]. The hand motor area was identified in all thirty T1w atlas images and followed down to the level of the cingulum, which was used as the inferior cut off of the precentral gyrus. Analysis of CBF in the primary motor cortex was performed in GM only.

#### CBF post-processing in the primary motor cortex

For every pCASL scan, mean GM CBF values were obtained from the left and right superior precentral gyrus (primary motor cortex). CBF values were averaged per session over the four FT blocks and over the four rest blocks, and then over the primary motor cortex bilaterally, such that for every session we obtained one mean GM CBF value in the bilateral primary motor cortex for the FT condition (CBF_FT_) and one for the rest condition (CBF_rest_, Fig 1C).

#### Task-induced regional CBF changes within sessions

To assess task-induced CBF changes in the primary motor cortex, CBF_FT_ and CBF_rest_ within sessions were compared with paired t-tests (p < .05). Absolute CBF changes as a result of finger tapping are referred to as delta CBF: [CBF_FT_−CBF_rest_] (Fig 1C).

#### Reproducibility of regional resting CBF and regional task-induced CBF changes

Intra- and inter-sequence reproducibility were assessed by the following measures (Fig 1C):

1. Within-subject coefficients of variation (wsCV) were calculated as the ratio of the standard deviation of the CBF difference (SD_diff_) between sessions to the mean CBF value of those sessions: wsCV = 100% (SD_diff_/mean value). The SD_diff_, rather than the SD of the mean, was used to reflect the extent of variability in differences in relation to the mean.
2. Mean CBF values over sessions, mean CBF differences between sessions, SD_diff_, and wsCVs and their 95% confidence intervals (CIs) are reported for CBF_rest_, CBF_FT_, and delta CBF.
  Intra-sequence measures were calculated between the two sessions per sequence. Inter-sequence measures were calculated by comparing GE (session) 1 to Philips 2 and GE 2 to Philips 1 (n = 44).
3. Intraclass correlation coefficients (ICC) and 95% CIs were calculated for CBF_rest_, CBF_FT_, and delta CBF. A two way-random model and absolute agreement were employed to allow for generalization of the results and to take into account systematic variability between sequences, respectively. ICCs were defined as function of ANOVA mean squares using the following formula [32]:
  $$\frac{BMS-EMS}{BMS+\left(k-1\right)EMS+k\;/\;n\left(JMS-EMS\right)}$$
  in which BMS refers to the between-targets mean square (i.e. variance between participants), JMS refers to the between-judges mean square (i.e. variance between intra- or inter-sequence sessions) and EMS to the residual mean square (i.e. residual sources of variance), in a two-way ANOVA with n = 22 (intra-sequence) or 44 (inter-sequence) targets and k = 2 judges.
  Inter-sequence ICCs were calculated between the two sessions per sequence by comparing GE (session) 1 to Philips 2 and GE 2 to Philips 1 (n = 44).
4. Bland-Altman plots and 95% limits of agreement (mean difference ± 1.96 SD_diff_) were created for CBF_rest_ and delta CBF to visualize agreement within and between sequences.

Statistical analyses were carried out in IBM SPSS Statistics, version 20.0 (New York, USA).

## Results

### Participant characteristics

Nine male and 13 female volunteers with a mean age of 22.1 ± 2.1 years (range: 19–27 years) participated in the study. It should be noted that one participant had CBF values that were 2–3 standard deviations higher than the group mean, but this participant was retained in the analysis as data were normally distributed (Kolmogorov-Smirnov tests did not detect significant deviations from normality in any session, p>.05). The two sessions scanned using the GE sequence were separated by 2.8 ± 1.0 weeks and those using the Philips sequence by 2.6 ± 0.9 weeks (not significant (n.s.)). Inter-sequence sessions were separated by 3.1 ± 1.1 weeks (GE 1 –Philips 2) and 2.6 ± 1.9 weeks (GE 2 –Philips 1), n.s. Both sessions scanned with the GE sequence took place at an earlier time of day than those scanned with the Philips sequence: 3:26pm ± 4h00min and 3:55pm ± 3h34min versus 8:16pm ± 2h06min and 7h47pm ± 2h38min respectively, p < .05.

### Whole-brain voxel-wise activation sensitivity of pCASL sequences

**Fig 2:**
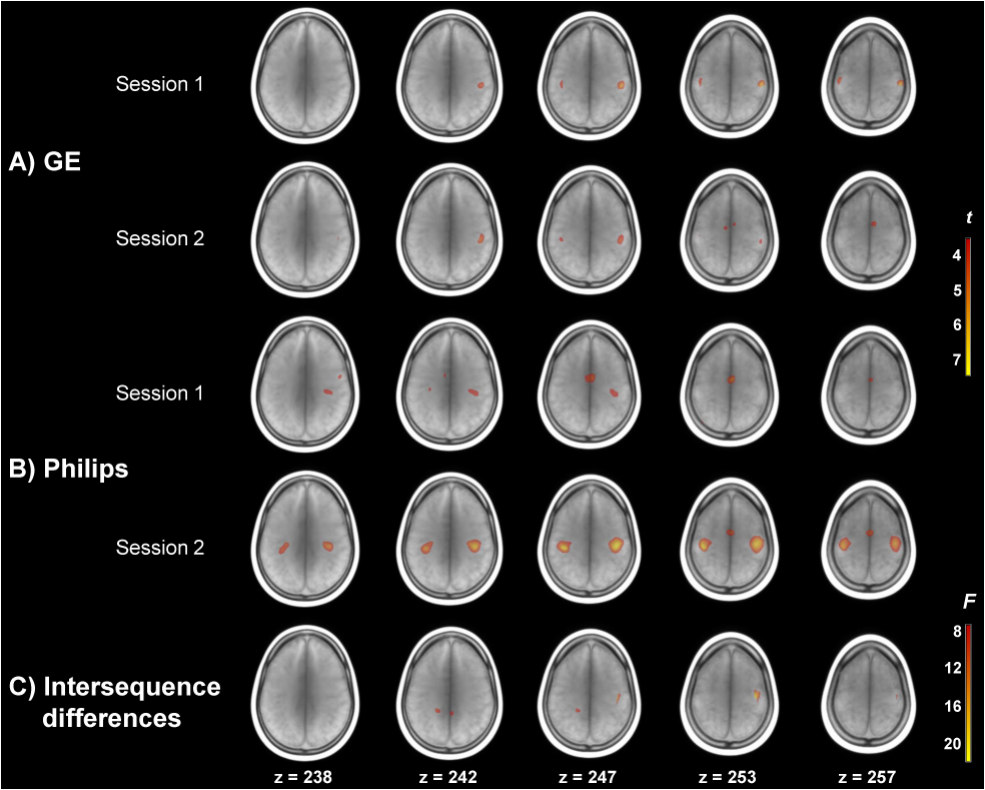
whole-brain voxel-wise CBF differences associated with finger tapping compared to rest. Activation maps are overlaid on a mean T1w scan. T-maps for the two sessions of A) GE and B) Philips sequences are thresholded at *t* = 3.52, p < .001 (uncorrected). C) shows the F-map depicting differences in activation between pCASL sequences, thresholded at *F*(2,63) = 7.7, p < .001 (uncorrected).

Voxel-wise CBF changes in relation to FT are illustrated with t-statistic maps in Fig 2. Both with GE (Fig 2A) and Philips (Fig 2B) CBF increases were observed in the bilateral primary motor cortex in both sessions. Additional activation was observed in the supplementary motor area and the left cerebellum in GE session 2; and in the thalamus, and supplementary motor area in both Philips sessions. As can be appreciated visually, Philips (Fig 2B) seems to be more sensitive to detect activation than GE (Fig 2A). Upon formal assessment with repeated measures ANOVA (Fig 2C), differences between pCASL sequences were found in the right primary motor cortex, left precuneus, right posterior cingulate, and in the bilateral thalamus.

### Task-induced regional CBF changes within sessions

Absolute CBF values differed systematically between pCASL sequences, with mean CBF_rest_ values being 4.1 mL/100g GM/min lower as measured with GE than with Philips. The increase in CBF in the motor cortex as a result of FT was significant for both GE sessions and the second Philips session (Table 3). The increase in CBF due to FT relative to CBF_rest_ was larger for the two GE sessions (6.13 and 4.27%) than for the two Philips sessions (2.27 and 3.53%).

**Table 3:** mean CBF and standard deviations (mL/100g GM/min) in the motor cortex during finger tapping and rest, and respective p-values; and relative CBF increase expressed as a percentage of resting CBF, per sequence per session.

	GE				Philips			
	Session 1		Session 2		Session 1		Session 2	
	FT	R	FT	R	FT	R	FT	R
**Mean**	65.8	62.0	66.2	63.5	69.6	68.1	67.8	65.5
**SD**	11.49	14.11	9.50	10.60	13.10	13.39	11.65	14.45
**p-value**	.005		.010		.078		.020	
**Relative CBF increase (%)**	6.13%		4.27%		2.27%		3.53%	

CBF: cerebral blood flow, SD: standard deviation, FT: finger tapping, R: rest.

### Reproducibility of regional resting CBF and regional task-induced CBF changes

WsCV, SD_diff_, mean CBF values and ICCs and 95% CIs are reported for CBF_rest_, CBF_FT_, and delta CBF in Table 4. WsCVs were comparable within and between pCASL sequences for CBF_rest_ and CBF_FT_.

**Table 4:** Mean CBF measurements and reproducibility estimates between sessions and sequences for resting CBF (CBF_rest_), finger tapping CBF (CBF_FT_) and delta CBF in the primary motor cortex.

		GE	95% CI	Philips	95% CI	Inter-sequence	95% CI
**CBF**_**rest**_	Mean CBF	62.7	57.6–67.8	66.8	61.0–72.6	64.8	61.1–68.5
	Mean CBF difference	-1.51	-5.87–2.85	2.58	-1.59–6.76	-4.08	-7.07 –-1.09
	SD difference	9.83	6.67–13.0	9.42	6.40–12.4	9.81	7.67–11.9
	wsCV (%)	15.7	9.68–21.7	14.1	7.55–20.7	15.1	10.9–19.4
	ICC	0.69	0.40–0.86	0.77	0.52–0.90	0.69	0.47–0.82
**CBF**_**FT**_	Mean CBF	66.0	61.7–70.2	68.7	63.6–73.9	67.4	64.2–70.5
	Mean CBF difference	-0.42	-4.32–3.47	1.81	-2.05–5.68	-2.76	-5.77–0.26
	SD difference	8.78	5.96–11.6	8.71	5.91–11.5	9.90	7.74–12.1
	wsCV (%)	13.3	8.20–18.4	12.7	6.81–18.5	14.7	10.9–18.5
	ICC	0.66	0.34–0.85	0.75	0.50–0.89	0.61	0.39–0.77
**Delta CBF**	Mean CBF	3.25	1.42–5.09	1.93	0.61–3.24	2.59	1.39–3.79
	Mean CBF difference	1.09	-1.54–3.72	-0.77	-3.30–1.77	1.32	-0.13–2.78
	SD difference	5.93	4.02–7.83	5.72	3.88–7.55	4.79	3.75–5.83
	wsCV (%)	182	180–185	297	294–299	185	183–186
	ICC	0.32	-0.10–0.65	0.04	-0.40–0.45	0.45	0.18–0.65

CBF: cerebral blood flow, SD: standard deviation, wsCV: within subject coefficient of variation, ICC: intraclass correlation coefficient, CI: confidence interval.

**Fig 3:**
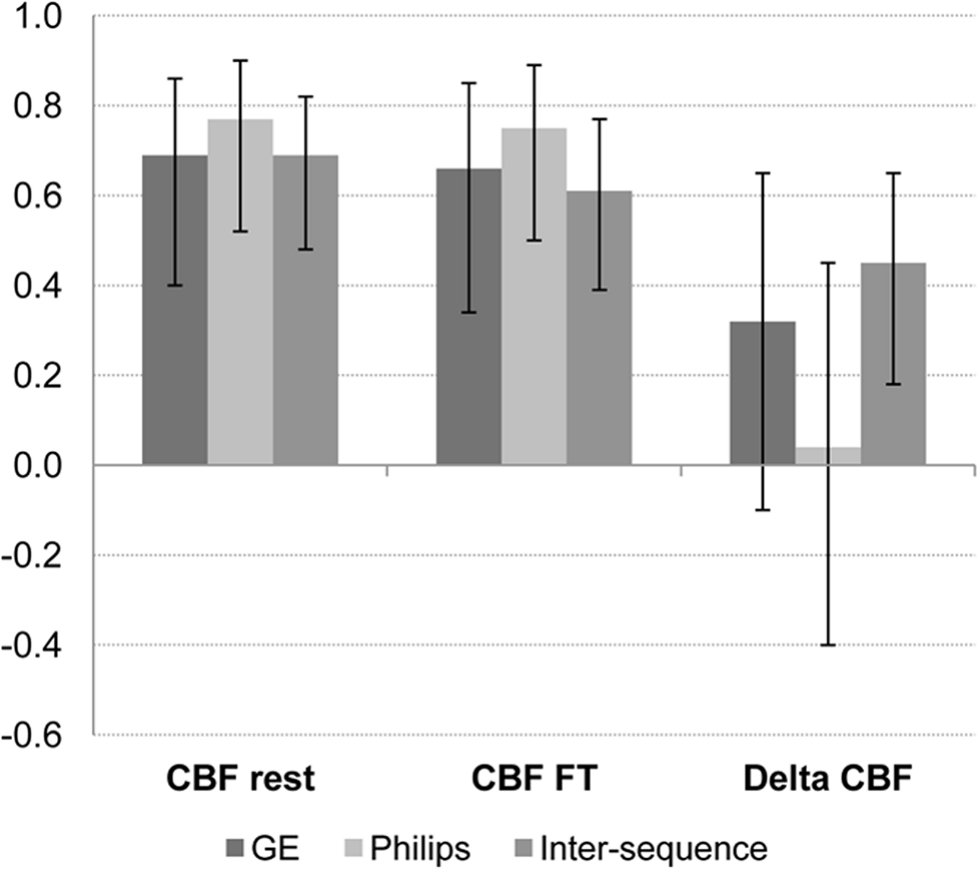
intra- and intersequence intraclass correlation coefficients for CBF_rest_, CBF_FT_ and delta CBF in the motor cortex. Error bars denote 95% confidence intervals.

Reproducibility of CBF_rest_ and CBF_FT_ in terms of ICCs was moderate to good for both sequences, with ICCs of .69 and .66 for GE and .77 and .75 for Philips, respectively (Fig 3). Reproducibility of absolute delta CBF was poor for both sequences with ICCs of .32 (GE) and .04 (Philips), and CIs being 1.5–2.3 times larger than for CBF_rest_ and CBF_FT_. Between sequences, reproducibility was reasonable for CBF_rest_ (.69) and CBF_FT_ (.61) and fair for absolute delta CBF (ICC: .45). Inter-sequence CIs for delta CBF were approximately 1.3 times larger than for CBF_rest_ and CBF_FT_.

**Fig 4:**
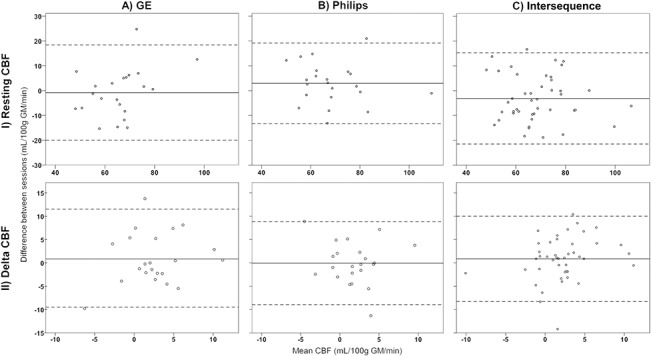
agreement within and between pCASL sequences for resting CBF and delta CBF. Bland Altman plots of agreement between the two sessions per sequence (A, B) and between sequences (C) for resting CBF (I) and delta CBF (II) in the primary motor cortex. The solid line indicates the mean difference between sessions, dotted lines the 95% limits of agreement.

Fig 4-I and 4-II show the agreement within and between sequences for CBF_rest_ and absolute delta CBF respectively. For both CBF_rest_ and delta CBF, differences within sequences were somewhat larger for measurements performed with GE (Fig 4A-I and 4A-II) than with Philips (Fig 4B-I and 4B-II). The low intra-sequence reproducibility of delta CBF with Philips in particular (Fig 4B-II) is illustrated by the variability relative to the mean effect being higher for delta CBF than for CBF_rest_ (Fig 4B-I). Both CBF_rest_ and delta CBF show a comparable spread in differences within and between sequences. Fig 4C-I and 4C-II show the agreement between sequences for CBF_rest_ and delta CBF respectively, and illustrate that although the spread in differences is higher for CBF_rest_ (Fig 4C-I), the variability relative to the mean effect is 1.5 times as large for delta CBF (Fig 4C-II).

## Discussion

Using pCASL product sequences as supplied by two MR vendors, we found that sensitivity to detect primary motor cortex activation was sufficient and comparable for both sequences. Secondly, we found intra- and intersequence reproducibility of resting CBF in the motor cortex to be reasonably good, as was reproducibility of CBF during finger tapping. More important in the context of fASL however, we found absolute CBF changes to be only moderately reproducible, both within and between sequences, despite a more or less consistent task-induced CBF increase within sessions at the group level.

This study adopted a pragmatic and clinically applicable approach in employing pCASL product sequences as supplied by the vendors. Parameters were adjusted to match each other as much as possible, but within the constraints of the provided sequence. This reflects the de facto situation of commercially supplied sequences in which parameters can only be adjusted to a certain extent. As CBF measured with ASL is claimed to be and promoted as a quantitative measure, one would assume that measurements are independent of sequence parameters. Here we show to what extent standard implementation of the sequences gives rise to substantial differences and thus limited reproducibility for functional ASL.

The increase in CBF in the motor cortex during finger tapping is in line with previous cross-sectional fASL studies employing finger tapping paradigms [5,7,33,34]. Our results replicate these findings within both sequences, except in one of the Philips sessions. Although we found the extent and spatial patterns of activation to differ between sequences, they both detected activation in the primary motor cortex as a result of finger tapping, and formal comparison of activation patterns between sequences demonstrated only minor differences in motor areas. This supports the notion that on the group level, fASL is sufficiently sensitive to detect activation in the primary motor cortex, and that differences in sensitivity between pCASL product sequences are acceptable. The magnitude of CBF changes was, however, surprisingly modest in our study compared to previous studies, which may–at least partially–be due to methodological differences. For instance, our ROI comprised the entire superior precentral gyrus, which may have resulted in only a modest increase since CBF is averaged over this entire ROI. Previous studies quantified CBF in ROIs more spatially specific to activation, such as the ‘precentral hand knob’ or activated volumes [5,35].

We found reproducibility of resting CBF in the motor cortex to be reasonably good within and good between pCASL sequences. Our intra-sequence results are in line with previous whole-brain resting CBF studies that have shown reasonable to good reproducibility within and between sessions on the same scanner [23], as well as between scanners of the same vendor [16,24]. We recently compared resting gray matter CBF between two different pCASL sequences and also found it to be well reproducible on the global level [25]. The current study focused on regional resting gray matter CBF and showed that, when compared to global CBF, reproducibility was slightly lower. Previous studies found that smaller regions are subject to higher variability and thus may yield lower reproducibility estimates [11,23]. In addition, regional reproducibility has been found to be lower for CBF measured at an interval of 2–4 weeks than for measurements within one day, implying that temporal physiological differences dominate between-weeks reproducibility [23,36]. Other sources of physiological variation may also be present, such as the one participant with CBF values that were consistently higher than the group mean. This physiological variation could have been accounted for by adding global CBF as a covariate, but as we aimed to demonstrate the variability in *absolute* regional CBF changes, scaling the signal would have defeated the objective of the current study.

Apart from temporal dynamics in physiology, regional variability could also be affected by the difference in effective post-labeling delay (PLD) between pCASL sequences. In order for labeled blood to reach the relatively superiorly located motor cortex, arterial transit time (ATT) is longer than for inferior regions, because of the larger distance between the labeling plane and the target tissue. This was previously demonstrated by Gonzalez-At *et al*. [37], who measured ATT to the visual region (5.1 cm from the labeling plane) to be 514 ms and to the motor region (11.5 cm from the labeling plane) 906 ms. Although in the current study the same initial PLD (1 525 ms) was applied for both sequences, the 2D multi-slice acquisition employed by Philips may have allowed labeled blood more time to reach superior slices, including regions with longer arrival times, than did the single time-point 3D acquisition employed by GE. This difference in effective PLD between sequences may have contributed to decreased reproducibility between their respective measurements. Moreover, the level of reproducibility of different regions seems to vary with PLD [16], with a PLD of 2 500 ms yielding better reproducibility than a PLD of 1 500 ms when using a 3D single time-point sequence. Therefore, effective PLD differences may have affected our results, as the precentral gyrus is located at the superior aspect of the brain, and therefore exhibits longer transit delays. This may have led to an underestimation of CBF values due to incomplete inflow of label, and to higher variability due to difference in arterial arrival times. Note that this is not resolved by merely prolonging the PLD, because although this may be a benefit for optimal bolus delivery, it also compromises signal due to label decay.

Differences between sequences may not only affect resting CBF measures, but also those of motor activation. Studies that compared fASL data obtained with 2D and 3D sequences found that activated clusters are generally larger when using 3D sequences, while 2D sequences yield larger effect sizes in terms of relative CBF changes [38,39]. Our results on the other hand showed larger activated clusters with the 2D sequence, and larger relative CBF changes with the 3D sequence. The larger effective PLD of Philips may have allowed more labeled blood to reach the primary motor cortex during finger tapping than GE, yielding larger activation clusters. In addition, the 3D sequence is more susceptible to spatial blurring, which obscures the gray matter to white matter contrast [38], and may attenuate signal from the gray matter. The larger relative signal change measured with GE on the other hand may be explained by decreasing ATT as a result of finger tapping [37]. Although (pseudo-) continuous ASL techniques are not very sensitive to changes (especially decreases) in ATT [40], it is likely that the known decreased ATT during neuronal activation [41] also leads to faster extravasation of the label into the tissue compartment, leading to faster decay of the label as the longitudinal relaxation time of tissue is shorter than that of blood. This would lead to an underestimation of CBF during activation. Additionally, because of its shorter effective PLD, particularly in the superior regions, such shorter ATT after finger tapping may have caused the relative signal change as measured by GE to be higher than by Philips.

The reproducibility of delta CBF, i.e. the CBF difference observed between finger tapping and rest, is less straightforward to interpret. Despite the smaller inter-session variation of delta CBF differences (as indicated by smaller standard deviations of differences) when compared to that of resting CBF and finger tapping CBF, wsCVs are extremely high. The relatively small effect of delta CBF is more likely to be susceptible to high variabililty than resting and finger tapping CBF, which may affect its reproducibility to a larger extent than the other CBF measures. This is in fact reflected by the ICCs indicating poor intra-sequence reproducibility. Previous fASL studies reported wsCVs of 10–11% [35,42], and ICCs up to 0.74 [43], between sessions that were a week apart. However, these studies assessed the reproducibility of relative CBF changes, instead of absolute CBF changes. It has been suggested that relative CBF changes are more accurate and robust than absolute CBF changes, as they may reduce potential effects of basal perfusion variations on measures of neuronal activation [43]. Relative CBF changes may therefore generate higher ICCs than absolute CBF changes. Nevertheless, as mentioned earlier, as absolute quantification is a specific advantage of fASL, it seems more appropriate to investigate the reproducibility of absolute CBF changes.

Slight variation in signal change as a result of finger tapping has been observed, but shown to be similar between sessions that took place on the same day or on different days [42]. Raoult *et al*. [43] found similar levels of variation using a finger flexion-extension paradigm. Moreover, they found task-induced CBF to be higher, albeit not significantly, with shorter sequence lengths, and concluded that a motor paradigm with 4 blocks of 30s on/off activation is optimal for clinical practice [35]. Longer sequence durations are considered to induce habituation and thus decreased activation. Our paradigm consisted of 2 minute blocks of on/off activation because of the limited temporal resolution of the GE sequence. These relatively long blocks thus may have attenuated activation, and thus the effect of finger tapping as compared to other studies, which may have reduced reproducibility.

On the other hand, one of the major reasons to use ASL for functional imaging is its suitability for low frequency designs, as it is much less sensitive to drift effects over time than BOLD fMRI [44]. Wang *et al*. [5] even demonstrated that fASL shows constant sensitivity across different task frequencies corresponding to blocks lengths ranging from 0.5–5 minutes, with ASL outperforming BOLD contrast at a block length of 4 minutes. Some higher cognitive functions, such as sustained attention [1], depend on an experimental design with even longer blocks to detect slow, low-frequeny signal changes of interest, for which fASL is particularly well suited. Despite ASL’s appropriateness for such cognitive paradigms, we purposely chose a simple behavioral paradigm known to elicit robust and consistent regional activation, before moving on to more complex processes and paradigms. We find that even this simple motor activation paradigm gives rise to substantial variability, which warrants caution with respect to more complex and less robust designs.

To our knowledge, no studies exist on the reproducibility of task-induced CBF changes using pCASL product sequences from two different vendors. In the current study, we found reproducibility of task-induced CBF to be comparable within and between sequences, both in terms of wsCV and ICC. Nevertheless, the findings indicate that absolute CBF changes in the motor cortex still vary considerably, and this variation needs to be taken into account when comparing regional quantitative CBF changes, particularly between sequences. Therefore, absolute fASL data should not be simply pooled between product sequences.

This study has some limitations. First, we quantified CBF using a model that simplifies the actual *in vivo* situation, which would have required measurement of many variables that are difficult to obtain on an individual basis. This is illustrated by the discrepancy between the results of the voxel-wise group analysis and region-wise analysis: whereas the qualitative voxel-wise analysis showed acceptable inter-sequence differences, results from region-wise quantitative analysis are far less similar. Nevertheless, this lends support to our conclusion that current standard implementation of ASL and recommended analysis of ASL are not–yet–suited for quantitative functional ASL experiments. Second, we did not collect information on motor behavior. Although variations in frequency were avoided by externally pacing the finger tapping, we may have missed individual variations in tapping, which may have added to the variability. Next, as time of acquisition differed between sequences, with GE data collected earlier on the day, diurnal fluctuations in CBF may have added to variability between sequences. This potentially affected reproducibility of resting CBF more than that of delta CBF, as the latter is based on a subtractive measure. Still, inter-sequence reproducibility of resting CBF was found to be reasonably good. Furthermore, subsequent analysis of the Philips data was performed in a similar manner to maximize comparability with the GE data, i.e. by averaging over rest and activation periods, whereas one would normally choose to exploit the higher temporal resolution in a more formal manner within the design matrix. Finally, due to practical constraints we studied the product sequences of only two out of the three major vendors on the market. Although assessment of variability between the three vendors would have been more comprehensive, the current study was conducted as a proof-of principle, and demonstrated as such that substantial variability is already evident when product sequences of two vendors are compared. Future work should be directed at optimizing ASL sequences for functional imaging, and at assessing sensitivity and reproducibility of fASL in single-subject designs, as longitudinal studies and clinical application of fASL will eventually need to be aimed at repeated measurements within individuals.

In conclusion, in a voxel-wise whole-brain analysis, fASL shows sufficient sensitivity to detect regional CBF changes on a group level, both within and between pCASL product sequences of two different vendors. The between sequence reproducibility of fASL is comparable with within sequence reproducibility, although inter-sequence differences in readout should be taken into account. Although reproducibility of regional resting CBF is affected by differences in sequence implementation, particularly in the readout, resting CBF in the motor cortex may provide a reasonably consistent baseline to compare task-induced CBF to. The relatively low reproducibility of task-induced CBF changes in the primary motor cortex, however, should be taken into consideration when comparing fASL data between sessions and particularly between pCASL product sequences as implemented by different vendors. Its interpretation should be performed with caution in repeated measurements and multicenter designs, as current vendor-specific implementations do not allow for simple pooling of functional ASL data.
